# PM534, an Optimized Target-Protein Interaction Strategy through the Colchicine Site of Tubulin

**DOI:** 10.1021/acs.jmedchem.3c01775

**Published:** 2024-01-31

**Authors:** Daniel Lucena-Agell, María José Guillén, Ruth Matesanz, Beatriz Álvarez-Bernad, Rafael Hortigüela, Pablo Avilés, Marta Martínez-Díez, Gema Santamaría-Núñez, Julia Contreras, Iván Plaza-Menacho, Juan F. Giménez-Abián, María A. Oliva, Carmen Cuevas, J. Fernando Díaz

**Affiliations:** † Unidad BICS. Centro de Investigaciones Biológicas Margarita Salas, Consejo Superior de Investigaciones Científicas, Ramiro de Maeztu 9, 28040 Madrid, Spain; ‡ PharmaMar S.A., Avda de los Reyes 1, Colmenar Viejo, 28770 Madrid, Spain; § Centro Nacional de Investigaciones Oncológicas (CNIO), Melchor Fernández Almagro 3, 28029 Madrid, Spain

## Abstract

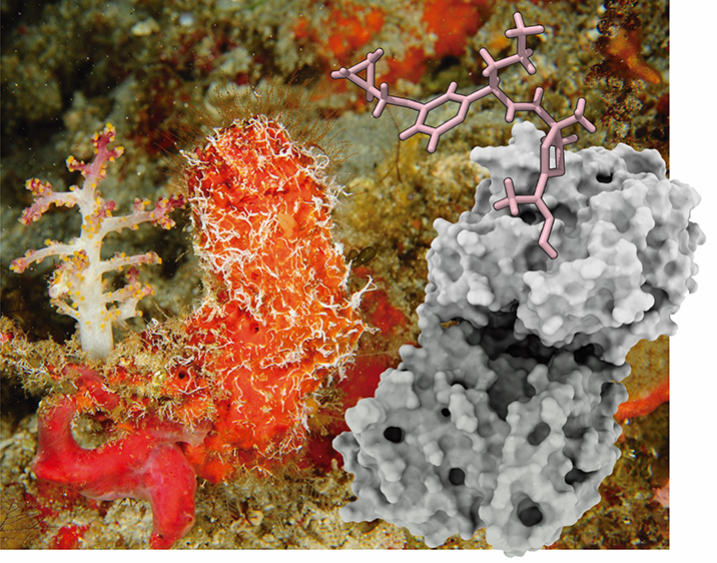

Targeting microtubules is the most effective wide-spectrum pharmacological strategy in antitumoral chemotherapy, and current research focuses on reducing main drawbacks: neurotoxicity and resistance. PM534 is a novel synthetic compound derived from the Structure–Activity-Relationship study on the natural molecule PM742, isolated from the sponge of the *order Lithistida*, family *Theonellidae*, genus *Discodermia* (du Bocage 1869). PM534 targets the entire colchicine binding domain of tubulin, covering four of the five centers of the pharmacophore model. Its nanomolar affinity and high retention time modulate a strikingly high antitumor activity that efficiently overrides two resistance mechanisms in cells (detoxification pumps and tubulin βIII isotype overexpression). Furthermore, PM534 induces significant inhibition of tumor growth in mouse xenograft models of human non-small cell lung cancer. Our results present PM534, a highly effective new compound in the preclinical evaluation that is currently in its first human Phase I clinical trial.

## Introduction

Despite recent relevant therapeutic approaches in cancer, advanced solid tumors remain mostly incurable, representing an urgent need for the development of new therapeutic options. Indeed, the demand for new agents with acceptable toxicities that improve the outcome of the current treatments for patients with advanced solid tumors is a top priority within the oncology field. Strikingly, microtubules are a major component of the cellular cytoskeleton and a highly attractive target for anticancer drug development due to their implication in cell division (chromosome segregation) and tumor vascularization, among other functions.^1^ These filaments grow from αβ-tubulin heterodimers, which are modulated by a wide range of small molecules known as microtubule-targeting agents (MTAs). MTAs contribute to the pharmacological regulation of tubulin deactivation and activation upon either the depolymerization or assembly of microtubules. So far, structural studies have highlighted up to eight different binding sites for exogenous molecules, namely colchicine,^2^ vinca,^3^ maytansine,^4^ pironetin,^5^ gatorbulin,^6^ and todalam^7^ for microtubule-destabilizing agents (MDAs); and, taxane^8^ together with laulimalide^9^ for microtubule-stabilizing agents (MSAs).

The first MTAs introduced for cancer treatment were vinca alkaloids in the late 1960s, followed 30 years later by taxanes.^10,11^ Since then, many MTAs have been developed for the treatment of numerous types of tumors, being the last included in the list, high-affinity compounds of the maytansine site that have been developed for acting as payloads in antibody–drug conjugates.^4,12^ Of particular interest is the colchicine binding domain (CBD), named after colchicine, an alkaloid extracted from *Colchicum autumnale*, which has been used as medicine throughout history and has allowed the isolation and characterization of tubulin.^13^ This domain sits mainly in β-tubulin, surrounded by the central hinge helices H7–H8, the strands of both tubulin β-sheets (N-terminal and intermediate domains) and, capped by two loops, the catalytic T7 loop of β-tubulin and the T5 loop of the α-tubulin subunit below.^14^ A wide variety of chemotypes target this domain (for a recent review see ref (15)) and based on the structural information available, Wang and co-workers proposed in 2016 a pharmacophore model for the colchicine domain consisting of five points, including three hydrophobic centers surrounded by two hydrogen bond centers.^16^ To date, compounds targeting the CBD interact either with the outer pocket of the domain, which involves the interface between β- and α-tubulin, or alternatively bind deeply in the β monomer, making no contacts with α-tubulin. In the first group, we find colchicine (with clinical applications limited by its toxicity, narrow therapeutic index, and its susceptibility to multidrug resistance that is mainly used on inflammatory diseases^17^), podophyllotoxin (used exclusively to treat genital warts and *Molluscum contagiosum* through topic administration due to gastrointestinal toxicity^18^), combretastatin (with several analogs under trials for cancer treatment but yet none on clinical use), and others (mainly derivatives and analogs of these)^19^ that have not reached clinics. In the second type of binders, there are benzimidazoles like mebendazole (anthelmintic^20^), nocodazole, plinabulin, etc. The only exception to this pattern of interaction is tirbanibulin (KX01) and its derivatives^21,22^ that bind along the whole domain (fulfilling three of the five centers of the proposed pharmacophore model).^16^ Recently, tirbanibulin has been relegated to the treatment of actinic keratosis due to low activity in clinical studies.^23^ Therefore, although a myriad of known compounds target the CBD,^24^ very few have reached clinics, and none have gotten to commercial phase for cancer treatment.

Nature is an inexhaustible source of continuous genetic evolution that produces novel compounds that have already been optimized over the years. In this sense, hundreds of biological compounds remain to be discovered, and the oceans have been revealed as an incredible source of biodiversity, with novel chemical structures and well-optimized mechanisms of action.^25^ In this work, we describe the discovery and development of PM534, a synthetic analogue of a natural compound originally isolated from the sponge *Discordemia* sp. that binds to the whole CBD, covering four of the five pharmacophore centers, and displays high tubulin binding affinity. Remarkably, we also have found that PM534 has a powerful antitumor activity in cells (lung epithelial and non-small lung cancer cells (NSCLC)) and, it is able to override typical resistance mechanisms against MTAs (overexpression of detox pumps or specific tubulin βIII isotype). Finally, this potent activity is also seen in a xenograft model of non-small cell lung cancer, highlighting an important difference with tirbanibulin, and pointing PM534 toward clinical trials as a promising new antineoplastic drug.

## Results

### PM534 Binds to Tubulin and Occupies the Whole Colchicine Domain

**Figure 1:**
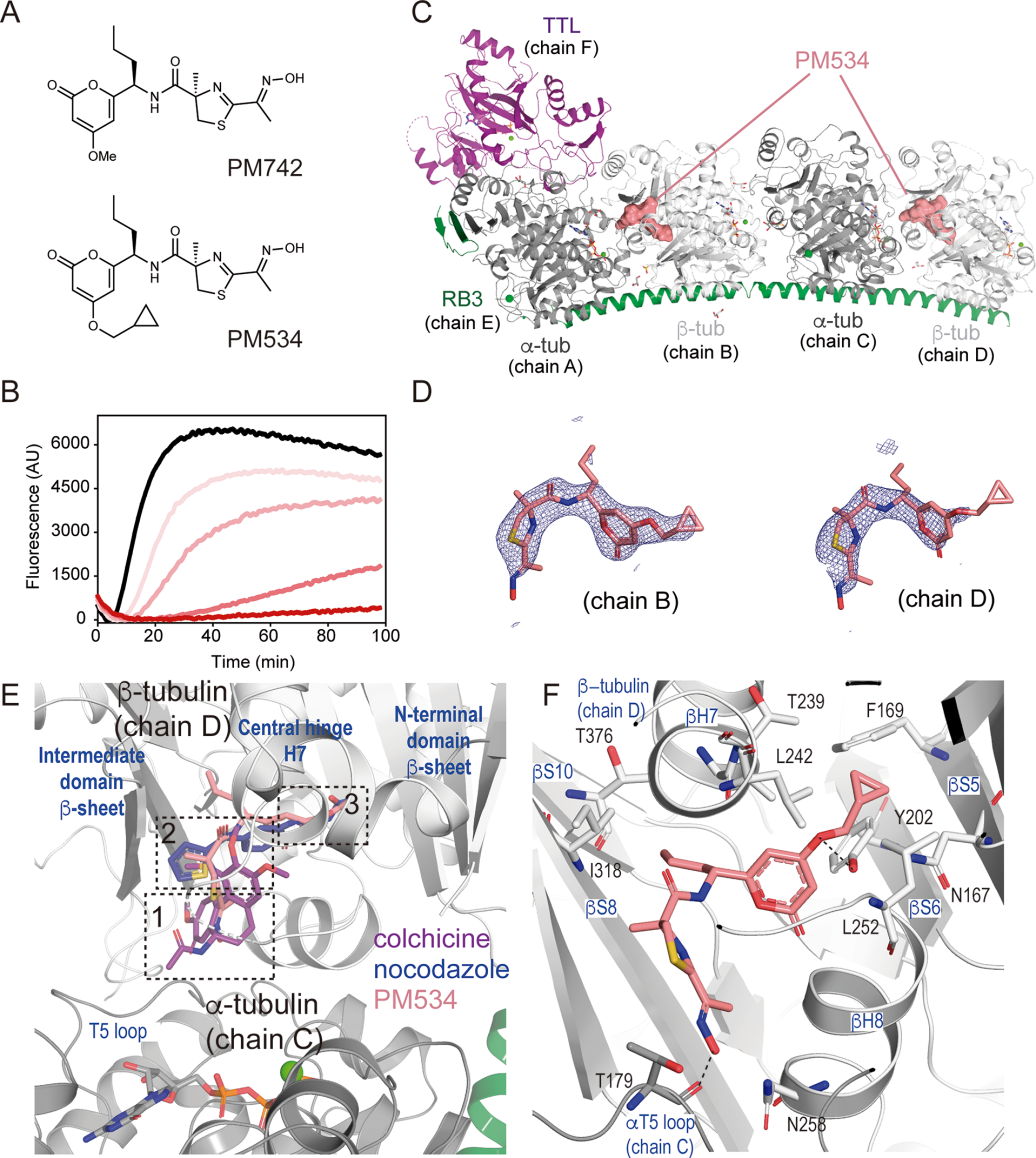
Crystal structure of PM534 bound to tubulin. (A) Chemical structures of PM534 and natural compound PM742. (B) Time-lapse polymerization assay of 18 μM tubulin in the presence of increasing PM534 concentrations (0.625, 1.25, 2.55, 5 μM, salmon color gradient) compared with control (0.5% DMSO, black line). (C) Overall structure of the T_2_R-TTL tubulin complex in the presence of PM534 (PDB 7ZYW), where proteins are in ribbon representation (α-tubulin in gray, β-tubulin in white, TTL in purple, and RB3 in green), and PM534 (salmon) is represented as surface bound to the colchicine domain of β-tubulin. (D) The sigma A weighted 2mFo-DFc (dark blue) electron density maps contoured at 1 sigma of PM534 (stick representation in salmon) at the colchicine domain of each of the protein chains, where it was identified. (E) Zoom into the colchicine domain in β-tubulin (ribbon representation chain D in white) showing colchicine (sticks purple), nocodazole (sticks blue), and PM534 (sticks salmon). The three zones of the pocket are labeled as dotted-line squares. (F) Zoom into the colchicine domain in β-tubulin (ribbon representation chain D in white), highlighting secondary structure elements involved in drug interaction and residues involved in hydrophobic and hydrogen bonding interactions (sticks representation).

PM534 (C_20_H_27_N_3_O_5_S, 421.51 Da) is a synthetic analog of PM742, a natural product obtained from a sponge, *Discoderma* sp. (order *Lithistida*) isolate (Figure 1A). In initial polymerization experiments, we found that the compound inhibits tubulin assembly in a dose-dependent manner (Figure 1B) so, we sought detailed structural information on the interaction of PM534 with tubulin using the T_2_R-TTL macromolecular complex, which includes two αβ-tubulin heterodimers in complex with one molecule of the stathmin-like domain of RB3 and one molecule of the tubulin tyrosine ligase.^26^ We soaked tubulin crystals with a concentrated solution of the compound and determined the T_2_R-TTL-PM534 complex structure to a 2.45 Å resolution (Table 1). We unequivocally found the PM534 ligand density at the CBD of both tubulin dimers within the T_2_R-TTL complex (chains B and D) (PDB entry 7ZYW), thus revealing in detail the interaction between the compound and tubulin (Figure 1C,D).

**Table 1:** Data Collection and Refinement Statistics for the T2R-TTL-PM534 Complex

	native T_2_R-TTL PM534
Data Collection	
space group	*P*2_1_2_1_2_1_
cell dimensions	
*a*, *b*, *c* (Å)	104.803, 158.026, 181.08
α, β, γ (deg)	90.00, 90.00, 90.00
resolution (Å)	49.656–2.45
*R*_meas_	0.100 (1.011)
*R*_merge_	0.080 (0.806)
*R*_pin_	0.044 (0.458)
CC_half_	99.2 (60.3)
*I*/σ(*I*)	4.0 (0.8)
completeness (%)	99.6 (100)
redundancy	4.7 (4.9)
Refinement	
resolution (Å)	49.656–2.45
no. of reflections	110332
*R*_work_/*R*_free_	0.2066/0.2364
no. atoms	34732
hydrogens	17103
water	119
average B-factors (Å^2^)	77.56
complex	77.86
ligand	69.97
solvent	54.38
Wilson B-factor (Å^2^)	58.85
r.m.s deviation	
bond lengths (Å)	0.002
bond angles (deg)	0.579
Ramachandran statistics (%) (allowed/favored/outliers)	97.57/2.43/0.00
rotamer outliers (%)	0.16
MolProbity overall score	0.92

Binding to the CBD implies the wedging at the αβ-tubulin intradimer interface, which blocks the curved-to-straight conformational transition that allows the activation of tubulin upon assembly into microtubules.^2^ However, the binding mechanism varies, and most of the compounds partially interact within specific regions of the CBD, which can be divided into three zones (Figure 1E): a central pocket (zone 2) and two accessory ones, with one facing the T5 loop of α-tubulin subunit below (zone 1) and the other buried deeper in β-tubulin and facing the β- sheet of the N-terminal domain (zone 3).^27^ These three zones include the five described centers of the pharmacophore model: zone 1 (hydrophobic center III and hydrophilic center V); zone 2 (hydrophobic centers I and II); and zone 3 (hydrophilic center IV). Compounds that interact with the outer pocket domain (like colchicine (PDB 4o2b), podophyllotoxin (PDB 5ca1), combretastatine (PDB 5lyj), or 7-aminonoscapine (PDB 6y6d)) bind to zones 1 and 2, and hence affect the hinge helices, the intermediate domain β-sheet, and the capped loops. Meanwhile, nocodazole (PDB 5ca1), mebendazole (PDB 7ogn), or plinabulin (PDB 5c8y) bind deeper in the pocket and occupy zones 2 and 3, therefore interacting with hinge helices and the N-terminal domain β-sheet. It is straightforward that, depending on the binding mode, the mechanism involved in blocking the curved-to-straight conformation of tubulin will slightly vary. Nevertheless, both types of compounds (those interacting with zones 1 and 2 and those interacting with zones 2 and 3) will directly affect central hinge helix H7, which is key to this assembly-linked tubulin conformational change.

Fallah-Tafti et al.^28^ described a synthetic non-ATP kinase inhibitor, which prevents tubulin polymerization by binding along the whole CBD and covering the hydrophobic centers I and II (βI318, βL248 and βI378) as well as the hydrogen bond center IV (βY202)^21^ of the pharmacophore model published earlier.^16^ Additional derived compounds have been shown to retain the interaction with these centers while making further contacts with α-tubulin T5 loop (αS178) and top of helix H7 (αR221).^22^ Our structure highlights that PM534 also interacts with the 3 zones of the CBD, making contacts with β-tubulin N-terminal domain (β-strands S5 and S6), helix H7, the intermediate domain (helixes H8 and β-strands S8 and S10,) and α-tubulin T5 loop (Figure 1F). PM534 cyclopropyl is deeply buried in the colchicine domain and forms mainly hydrophobic contacts with several residues of β-tubulin, including βN167 and βF169 (S5), βT239, and βL242 (H7) and βL252 (H8). The pyran ring establishes further interactions with βV238 (H7) and, the propyl chain with βI318 (S8) and βT376 (S10). Finally, the oxime moiety makes contacts with βN258 (H8) and α-tubulin T5 loop (αT179). Remarkably, despite a full occupancy of the compound at the site, the T5 loop was very floppy (especially in chain B), which may indicate a high fluctuation in the hydrogen bond established through this region. Otherwise, PM534 also hydrogen bond βY202 (S6) through the ether moiety and βN258 (H8), which further stabilizes the interaction of the compound with β-tubulin N-terminal domain. Therefore, considering the pharmacophore model, PM534 covers the hydrophobic centers I and II (βI318 and βI378) and the hydrophilic center V (αT179), and additionally, makes further hydrogen bonds, including centers IV (βY202) and V (βN258).

Likewise, PM534 interaction did not affect the overall curved conformation of tubulin, showing an rmsd of 0.299 Å over 378 Ca-atoms with the related apo-structure (PDB entry 4I55), similar to other CBD-targeting compounds. Furthermore, the structure displayed a conformation similar to that observed in the presence of other CBD compounds showing rmsd of 0.233 Å (over 396 Ca-atoms) with colchicine (PDB 4o2b), 0.295 Å (over 389 Ca-atoms) with 7-amino-noscapine (PDB 6y6d), 0.359 Å (over 382 Ca-atoms) with podophyllotoxin (PDB 5xlt), 0.259 Å (over 377 Ca-atoms) with plinabulin (PDB 5c8y), 0.256 Å (over 369 Ca-atoms) with nocodazole (PDB 5ca1) or, of 0.256 Å (over 369 Ca-atoms) with mebendazole (PDB 7ogn) among other CBD-targeting compounds. This high similarity denotes that the compound binding at the CBD does not significantly modify tubulin curvature, and their main mechanism of action involves a mechanical barrier that prevents the movement of secondary elements necessary to reach a specific structural conformation.

### PM534 Displays High Affinity and Slow Dissociation from the Colchicine Domain

**Figure 2:**
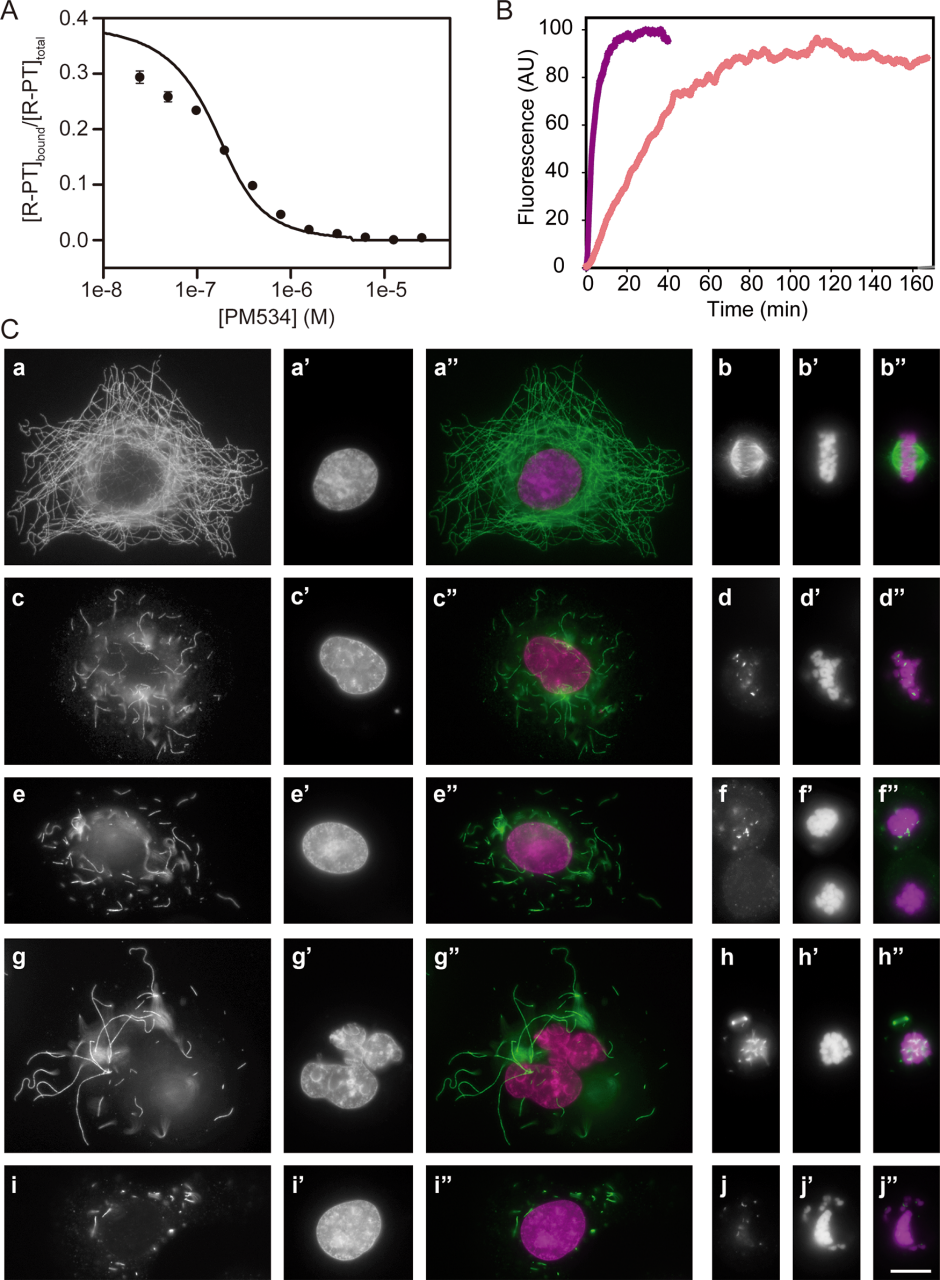
PM534 binding, dissociation kinetics, and effect on microtubular network. (A) Displacement of the fluorescent probe R-PT (0.2 μM) bound to tubulin (0.2 μM) by PM534 at 25 °C. The solid line was generated with the best fit (EQUIGRA 5.0) value of the binding equilibrium constant of the competitor, assuming a one-to-one binding to the same site. Data are the mean value of three experiments ± SD. (B) Dissociation kinetics of PM534 from tubulin at 37 °C (salmon line, average of 3 curves). Purple line shows the association kinetics of 60 μM colchicine to 2.5 μM tubulin in the absence of PM534. (C) Effects of PM534 on A549 cells. Each cell triplet shows α-tubulin immunostaining (DM1A antibody), the DNA labeling (DAPI staining), and the merge of the previous ones (tubulin in green and DNA in magenta). Panels a, c, e, g, and i are interphase cells, while panels b, d, f, h, and j correspond to mitotic cells. Panels a and b (0.5% of drug vehicle, DMSO); c and d (100 nM colchicine); e and f (50 nM podophyllotoxin); g and h (1 nM PM534) and; i and j (2 nM PM534). f panels show two cells, the upper one with a less severe phenotype than the bottom one. Scale bar: 10 μm.

To determine the binding affinity of PM534 to tubulin, we initially performed a competition assay using 2-methoxy-5-(2,3,4-trimethoxyphenyl)-2,4,6-cycloheptatriene-1-one (MTC), which is a *bona fide* medium affinity probe (4.7 × 10^5^ M^–1^)^29^ of the CBD.^30^ PM534 induced the full displacement of the probe at equimolecular concentrations, confirming its binding to the colchicine site. However, PM534 affinity is much higher than that of the probe, and hence, we required a higher affinity probe of the same site to determine PM534 binding affinity. To this end, we used (R)-(+)-ethyl 5-amino 2-methyl- 1,2-dihydro-3-phenylpyrido [3,4-*b*]pyrazin-7-yl carbamate (R-PT), with an affinity of 5.1 × 10^6^ M^–1^.^31^ At 25 °C, PM534 shows a binding affinity of 5.1 ± 0.3 × 10^7^ M^–1^ (*K*_d_ = 20 ± 1 nM, Figure 2A), (nearly 50 times higher than this of the natural product PM742 1.0 ± 0.1 × 10^6^ M^–1^, *K*_d_ = 960 ± 25 nM), which is the highest binding affinity for a derived natural compound of the CBD measured to date (colchicine, *K*_a_ 6.7 × 10^5^ M^–1^, *K*_d_ 1.5 μM;^32^ podophyllotoxin, *K*_a_ 1.9 × 10^7^ M^–1^, *K*_d_ 50 nM^33^ and; combretastatin A4, *K*_a_ 7.1 × 10^6^ M^–1^, *K*_d_ 140 nM^34^). Only the highly optimized compound 27 in ref (35) displays a higher affinity to the colchicine domain (*K*_a_ 3 ± 1 × 10^8^ M^–1^, *K*_d_ 3 nM).

Considering that in the case of colchicine, the in vivo efficiency comes from the very high dwell time at the CBD (*t*_1/2_ = 9.6 h),^36^ rather than from the binding affinity, we next sought the dissociation kinetics of PM534. We found that the compound displays a slow dissociation of the CBD (Figure 2B, *k*_off_ = 5.6 ± 0.1 × 10^–4^ s^–1^) that involves a retention half-time of 19.0 ± 0.3 min. Indeed, PM534 has a dissociation 30 times quicker than colchicine and 10.000 times slower than the reversible ligand 2-methoxy-5-(2′,3′,4′-trimethoxyphenyl) tropone (*k*_off_ = 6 ± 1 s^–1^, *t*_1/2_ = 0.12 ± 0.02 s),^37^ which is an analog of colchicine that has been deprived of the middle “B ring” but is still effective in blocking cell division.^38^ Compared to other MTAs used in clinics, PM534 retention time is 160 times higher than that of paclitaxel, (*k*_off_ = 9.1 ± 0.6 × 10^–2^ s^–1^, *t*_1/2_= 7.6 ± 0.5 s^39^), a compound that shows a reversible effect in cells.^40^

Finally, we approached the effect of the compound on the cellular microtubular network in order to determine if these kinetic features involved a better performance on cells. Immunofluorescence assays in lung carcinoma A549 cells (Figure 2C) showed that PM534 induces a complete disorganization of the tubulin cytoskeleton during interphase, with a significant reduction on the number and length of microtubules. During division, the compound also abrogated the formation of the mitotic spindle, generating a cell phenotype of hypercondensed chromatin and an aberrant chromosome distribution. These features correlate with the effect of MDA. Remarkably, only 1 nM PM534 is enough to induce a cellular landscape similar to that observed with 50 nM podophyllotoxin or 100 nM colchicine, which also agrees with the higher affinity and retention time measured.

### PM534 Elicits a Strong In Vitro Antineoplastic Activity

The very high affinity of PM534 and the results from the immunofluorescence assays suggest a high cytotoxicity of the compound. Consequently, we tested the antiproliferative activity of PM534 in four NSCLC cell lines: A549, Calu-6, NCI-H23, and NCI-H460. We analyzed cell viability with MTT and, upon cell treatment, we found a mean GI_50_ value of 2.2 ± 0.1 × 10^–9^ M, which is remarkably higher than that of colchicine (6 ± 1 x10^–8^ M) or vinblastine (1.1 ± 0.2 × 10^–7^ M), another MDA of the vinca domain. This means that in the same cell, it is necessary to reach 4 times (colchicine) or 200 times (vinblastine) higher concentrations to obtain an alike antiproliferative effect.

**Figure 3:**
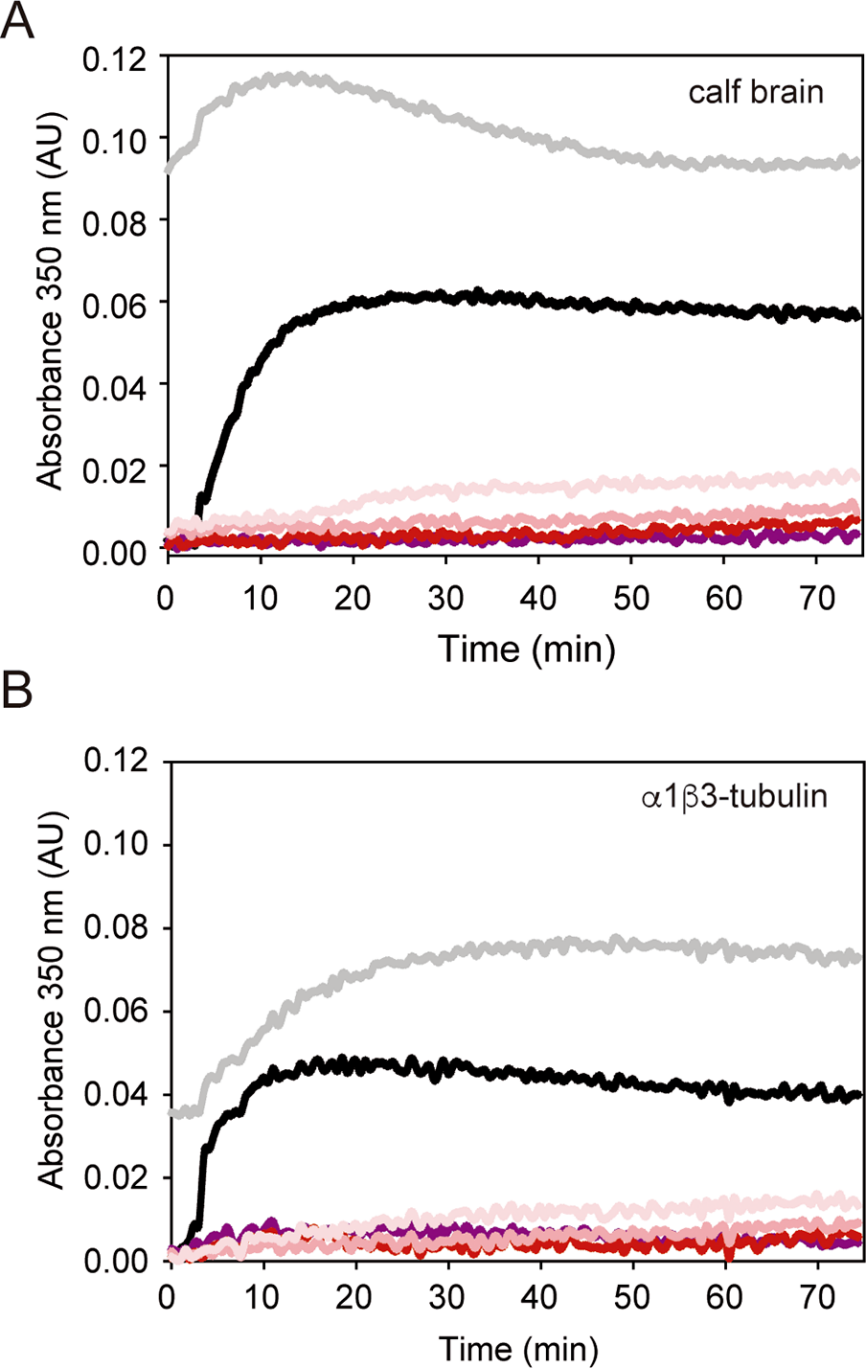
PM534 inhibition of brain tubulin vs βIII isotype. Time course assembly of 25 μM bovine brain tubulin (A) or recombinant human α1β3-tubulin (B) in the presence of DMSO (vehicle; black line), 27.5 μM paclitaxel (gray line), 27.5 μM podophyllotoxin (purple line), and increasing concentrations of PM534 (5 μM, 10 μM and, 27.5 μM, salmon color gradient).

MTAs constitute an important class of compounds in chemotherapy, but their clinical success has been compromised by the emergence of drug resistance, which is derived from several mechanisms that can be de novo or acquired. Two main different resistance mechanisms have been associated with the tubulin-targeted resistant phenotype in cancer patients: the overexpression of ATP-binding cassette family of proteins such as P-glycoprotein (P-gp) and alterations in the expression of tubulin isotypes.^41^ We approached the ability of PM534 to overcome these two typical cell resistant mechanisms using two isogenic pairs with a resistant counterpart to MTAs: A2780/A2780AD for P-gp overexpression and, HeLa/HeLaβIII for tubulin βIII isotype^42,43^ (Table 2). We found that despite PM534 showing similar activity as colchicine in A2780 cells, our compound worked far better in its multidrug-resistant counterpart A2780AD. When compared to podophyllotoxin, PM534 exhibits similar antiproliferative activity between the parental cells and their resistant counterparts, presenting resistance (*R*/*S*) indexes of 2.3 (P-gp overexpressing) and 0.8 (βIII-tubulin overexpressing). Remarkably, we found that PM534 also inhibited tubulin βIII isotype assembly in vitro (Figure 3). As expected, α1β3-tubulin demonstrates lower assembly propensity compared to bovine brain tubulin, which contains only 25% of βIII-tubulin^44^ and exhibits reduced susceptibility to the effects of paclitaxel.^45^ However, PM534 shows equal inhibitory effects on both tubulin sources, indicating that the change of Cys239 to Ser239 in the colchicine binding site does not confer resistance to the compound.

**Table 2:** Comparative Cytotoxicity (IC_50_) of Tubulin Targeting Agents^a^

	A549	A2780	A2780AD	*R*/*S*	HeLa	HeLa βIII	*R*/*S*
PM534	1.5 ± 0.1	1.4 ± 0.1	3.2 ± 0.3	2.3	1.6 ± 0.2	1.3 ± 0.2	0.8
PM742	23 ± 1	22 ± 1	54 ± 2	2.5	19 ± 1	28 ± 1	1.5
podophyllotoxin	16 ± 2	15 ± 1	20 ± 1	1.3	15.5 ± 0.4	18 ± 1	1.2
colchicine^b^	49 ± 5	15 ± 2	537 ± 50	35.8	11 ± 1	19 ± 1	1.7
compound 27^b^		23 ± 2	39 ± 15	1.7			
paclitaxel	5 ± 1	3.4 ± 0.6	392 ± 20	167	1.1 ± 0.2	20 ± 6	18
docetaxel	18 ± 5	3 ± 1	109 ± 10	38	0.7 ± 0.3	13 ± 1	18
vinblastine	1.5 ± 0.4	1.5 ± 0.2	70 ± 6	47	0.54 ± 0.08	4.4 ± 0.7	8.1
maytansine	0.59 ± 0.04	0.74 ± 0.08	19 ± 2	26	0.33 ± 0.04	1.1 ± 0.2	3.3

a IC_50_ values are in nM units. Data are mean values of three experiments ± SE.

b Data from ref (35).

It is worth mentioning that PM534 has only 2.5 times higher affinity than podophyllotoxin, but we have found that it displays about 10 times more cytotoxicity, which denotes higher efficiency in the inhibition of tubulin polymerization. Finally, despite the lower affinity, our results show that PM534 exhibited higher cytotoxicity than the highly optimized compound **27**.^35^ Moreover, PM534 has antiproliferative activity in the nanomolar range as MTAs in clinical use, targeting other sites in tubulin (paclitaxel, docetaxel, vinblastine, and maytansine). However, the performance of PM534 in the resistant cell lines is better.

### PM534 In Vivo Reduces Tumor Growth in NCI-H460 Xenografts

We finally pursued the effect of PM534 in a human NSCLC NCI-H460 cell line-derived xenograft and found that our compound showed a dose-dependence potency in vivo, increasing mouse survival with no signs of relevant systemic toxicity.

**Figure 4:**
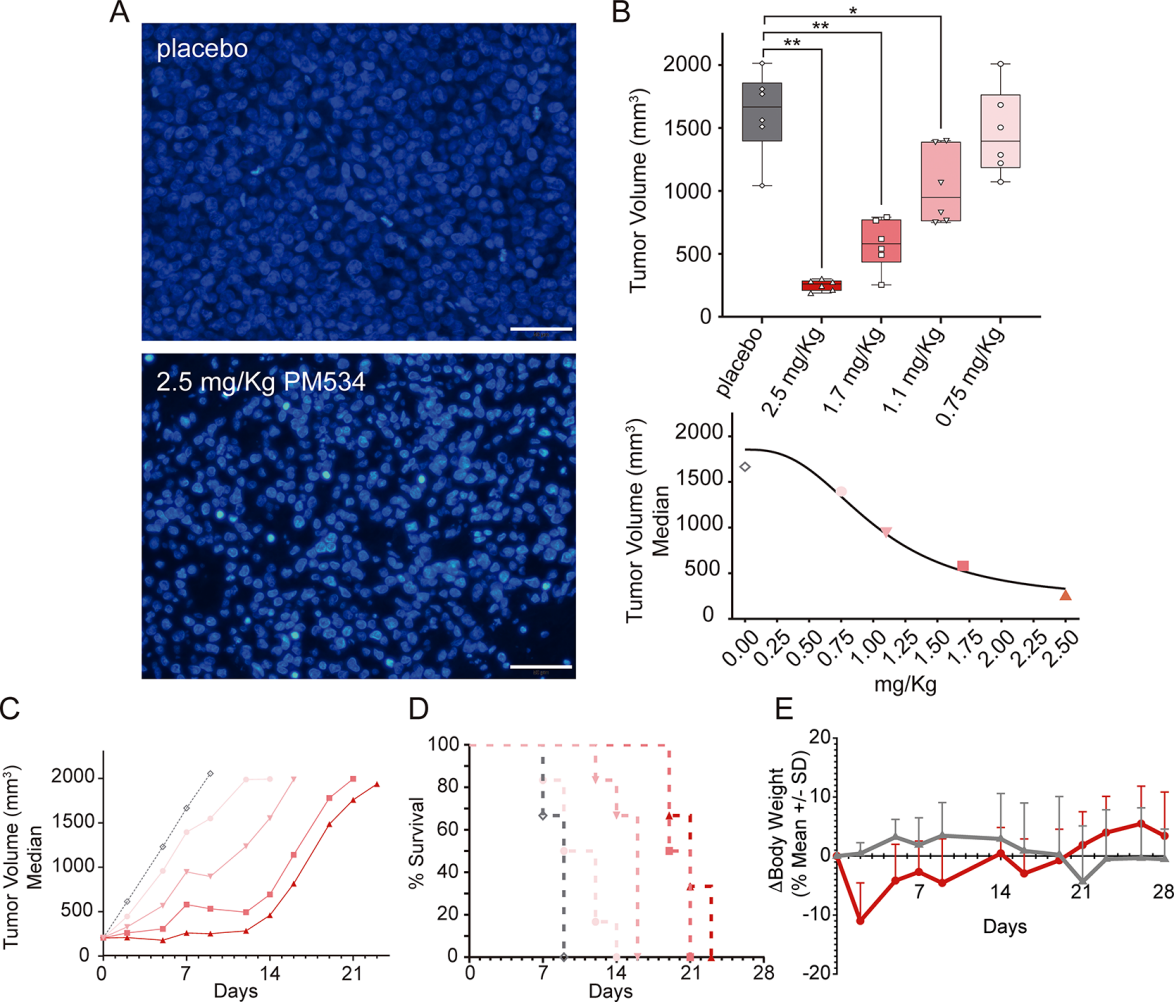
Antitumor effect of PM534 on NCI-H460 xenografts. (A) Representative DNA staining from tumor xenografts cells with Hoechst 33258, 24 h post placebo (top) or post administration of 2.5 mg/kg of PM534 (bottom), highlighting the increase of apoptosis in PM534-treated NCI-H460 tumors (magnification 40X and scale bar: 50 μm). (B) Box-and-Whisker Plot (top) and dose–response curve (bottom) of tumor volume on Day 7 after single administration of PM534 (on Day 0) in mice bearing NCI-H460 xenografts. (C) Tumor growth curve for mice bearing NCI-H460 xenografts treated with PM534 (on Days 0, 7 and 14). (D) Kaplan–Meier survival curve in mice bearing NCI-H460 xenografts and treated with a placebo or PM534. Color code in these graphs is: placebo (gray) and doses of mice treatment 0.75, 1.1, 1.7, and 2.5 mg/kg (salmon color gradient). (E)Body weight change in the experimental groups of mice bearing NCI-H460 xenographs treated with placebo (gray) or 2.5 mg/kg PM534 (salmon). Lines represent mean weights ± SD (*n* = 6).

We initiated the treatment of animals bearing NCI-H460 tumors once these reached a volume of ca. 200 mm^3^, and at 24 h, the number of apoptotic nuclei from harvested tumors of the placebo group was significantly lower (1.0 ± 0.1) than those from mice treated at the highest dose (2.5 mg/kg) of PM534 (10.0 ± 0.6, *p* = 0.0079) (Figure 4A).

In addition, we found that the treatment with PM534 resulted in a statistically significant reduction of the size of tumors at any dose except at the lowest dose (0.75 mg/kg), when compared to placebo-treated mice (Figure 4B, top). Indeed, our results highlighted that this compound displays a dose-dependence on the reduction of the volume of the tumors, and at day 7, immediately before the second dose, median tumor volume in mice treated with 0.75, 1.1, 1.7, and 2.5 mg/kg was 1395, 947.3, 580, and 260.4 mm^3^, respectively (Figure 4B, bottom). Finally, after having received the two weekly doses, overall, animals bearing NCI-H460 xenografts and treated with PM534 experienced a high statistically significant increase in the survival time (*p* = 0.0012) as compared to the placebo-treated group, except in mice administered with the lowest dose (0.75 mg/kg). While we sacrificed animals in the placebo group between days 7 and 9 (due to either tumor volume >2000 mm^3^, tumor necrosis, or both), median survival time was 10.5, 16, 20, and 21 days for mice treated with 0.75, 1.1, 1.7, and 2.5 mg/kg of PM534, respectively (Figure 4C,D). Remarkably, we did not observe any clinical signs of systemic toxicity after administration at the maximal tolerated dosis apart from a mean body weight reduction (nadir ca. 11.2% on Day 2), recovered afterward (−4.5 and −2.9% on days 5 and 7, respectively). After the compound administration on 7 and 14, similar pattern, although less marked, was recorded (Figure 4E).

## Discussion and Conclusions

The microtubule system is a well-validated target for the development of anticancer drugs.^11^ Controlled dynamics of microtubule assembly and disassembly are essential for both cell division and angiogenesis, two processes required by neoplastic tissues to grow. MTAs are able to perturb this delicate control, leading to rapidly proliferating cells to death and hampering endothelial cells to develop new blood vessels. However, the treatments using MTAs face several problems related with chemical instability, poor bioavailability, toxic side effects, and drug resistance phenotypes, the last being mainly associated to an increment of membrane detoxification pumps (e.g., P-gp overexpression)^46^ or to a differential expression of less sensitive tubulin isotypes (e.g., tubulin βIII isotype).^47^ It is therefore needed to develop compounds based on new, more stable chemotypes with different mechanisms of action that are able to overcome these problems, especially those related to the drug resistance phenotypes as compounds with high affinity for tubulin, which cannot be pumped out of the cell by the P-gp^48^ or compounds able to selectively target specific tubulin isotypes.^49^

PM534 targets the CBD of tubulin with a binding affinity in the nanomolar range and a slow kinetic dissociation constant (on the order of minutes), which justify the effect found in cells and in vivo. CBD-targeting compounds share a common mechanism on tubulin destabilization by blocking the curved-to-straight conformational transition necessary for tubulin activation upon assembly.^2^ However, there are differences in the binding mode that directly affect the kinetic properties of the compound and should explain cytotoxic differences found among known drugs targeting this site. Indeed, apparently the most effective compounds on cytotoxicity are very frequently those binding to zones 1 and 2 (Figure 1E). Podophyllotoxin displays a similar binding affinity and *R*/*S* index to PM534. Nevertheless, the high retention time of PM534 leads to a strong in vitro cytotoxicity, and is likely the reason for overcoming the effect of P-gp overexpression, a well-known mechanism of decreased colchicine-induced cytotoxicity in cells.^35^ Noticeably, we have found that PM534 is also effective in tumor cells that upregulate tubulin βIII isotype, as it is not affected by the Cys239 to Ser239 change in the colchicine binding site. This is a known resistance mechanism emerging as a feature of aggressive and treatment-refractory cancers.^41,45,50^ Notice that some of the compounds that bind deeper in the CBD are more selective for certain tubulin isotypes^51^ or bind preferentially to tubulins of other species.^52^ This ability to choose isotypes comes from some residues within the domain, like βC241 (in central hinge helix H7) and βI318 (pharmacophore center I), which are often involved in protein–drug interactions and are changed in the βIII-tubulin isotype to βS241 and βV318, respectively. PM534 does not interact with βC241, although it can establish hydrophobic contacts with βI318, which could work against overexpression of the tubulin isotype βIII. Nonetheless, we propose that PM534 can retain a high binding affinity to the tubulin isotype βIII because this compound binds to the whole domain, covering zones 1, 2, and 3 (Figure 1E), thus approaching the ideal pharmacophore model proposed by Wang et al.^16^ Moreover, PM534 covers a high number of centers, which most likely can explain the high retention time found (ca. 20 min). Finally, our in vivo data showed that PM534 causes an increase in the number of apoptotic nuclei very soon after treatment (24 h). This effect led to a dose-dependent reduction in tumor volume as well as an increase in the survival time of NSCLC xenograft-bearing mice treated with PM534, which is consistent with the mechanism of action.

In summary, our results demonstrate that PM534, a novel molecular structure, presents an extremely optimized interaction with the CBD that, at least in vitro, overcomes two of the most relevant mechanisms associated with tubulin-binding agent resistance (overexpression of P-gp^46^ and tubulin isotype βIII^47^). The very strong in vitro activity of PM534 successfully translates to an in vivo model, which makes this compound a potential candidate for the treatment of refractory tumors. PM534 has proved effective and safe in the preclinical evaluation and currently being developed in its first human Phase I clinical trial (REEC - Registro Espaol de Estudios Clnicos (aemps.es); ID: 2022-002032-31), initiated in 2022.

## Experimental Section

### Proteins and Chemicals

Purified calf brain tubulin and chemicals were obtained as previously described.^53^ The stathmin-like domain of RB3 and the chicken TTL protein preparations were done as described previously.^2,54^ 2-Methoxy-5-(2,3,4-trimethoxyphenyl)-2,4,6-cycloheptatrien-1-one (MTC) was a kind gift from Prof. Wei-Shuo Fang (Institute of Materia Medica, Beijing) while, (R)-(+)-ethyl 5-amino 2-methyl- 1,2-dihydro-3-phenylpyrido[3,4-*b*]pyrazin-7-yl carbamate (R-PT) was a kind gift of Prof. G.A. Rener, Organic Chemistry Research Department, Southern Research Institute, Birmingham, Alabama (Temple 1989). PM742 and PM534 are new chemical entities described in patent application: Martin, M. J.; Rodriguez-Acebes, R.; Cruz, P. G.; Francesch, A. M.; Cuevas, C. Anticancer Compounds. WO 2020127194, June 26, 2020. The patent describes the isolation of PM742 as well as the total synthesis of PM742 and PM534. PM534 was synthesized as described in the patent with a purity of >95% (Supporting Information). Colchicine, vinblastine, and podophyllotoxin were purchased from Sigma (#V1377 and #C9754).

### α1β3-Tubulin Expression and Purification

The plasmid pFastBAC Dual containing a codon-optimized DNA coding sequence for human α1/β3-tubulin^55^ was transformed in *E. coli* DH10 Bac competent cells. After transformation, cells were spread in plates with 50 μg/mL kanamycin, 7 μg/mL gentamicin, 10 μg/mL tetracyclin, 100 μg/mL Bluo-Gal, and 40 μg/mL IPTG and incubated 48 h at 37 °C. Plasmidic DNA (pDNA) was extracted from white colonies using E.Z.N.A. plasmid DNA Mini Kit I following the supplier instructions. To check pDNA insertion into the bacmid, a PCR reaction was performed using the M13 forward and M13 reverse primers. Protein was expressed in Sf9 insect cells^56^ with modifications. Briefly, Sf9 cells were seeded in a 6 well/plate at 0.5 × 10^6^ cells/ml in complete Sf900II media with 20 μg/mL of gentamycin. Cells were incubated for 30 min at 27 °C to allow the adhesion to the plate. The mix of transfection was prepared using 5 μL of bacmid (independently of the concentration) and 5 μL of the transfection reagent FuGENE HD (ratio 1:1) in 1 mL of Sf900II media without antibiotics. This mix was incubated at room temperature for 30 min. Cell medium was removed to add the mix of transfection, and then, cells were incubated overnight at 27 °C. After that, 1.25 mL of complete Sf900II media was added to the cells, and they were incubated for 5–7 days at 27 °C. The supernatant (p1) was used to inoculate 25 mL of suspension culture at (1–2) × 10^6^ cells/ml. This culture was incubated for 3–4 days at 27 °C with smooth shacking (92 rpm/min). The supernatant (p2) was used to inoculate 50 mL of suspension culture at (1–2) × 10^6^ cells/ml at 1:100 dilution. The culture was incubated in the above-mentioned conditions. Finally, cells were pelleted and used to purify the recombinant protein. A pellet of inoculated Sf9 cells (2.5 × 10^9^ cells) was defrosted on ice and resuspended in lysis buffer (400 mM Pipes pH 6.8, 5 mM MgCl_2_, 5 mM EGTA, 50 mM imidazole, 100 mM KCl, 0.2 mM GTP, 1 mM β-mercaptoethanol, and one tablet containing a protease inhibitor cocktail and benzonase). Cells were lysed using a manual homogenizer (50 pulses per 7 mL of crude lysate). Crude cell lysate was clarified by centrifugation using a JA-25.50 rotor at 16000 rpm, 45 min at 4 °C. The clarified supernatant was incubated with Ni-NTA beads for 1.5 h rotating at 4 °C. Beads were washed two times with three different buffers; dilution buffer: 400 mM Pipes pH 6.8, 5 mM MgCl_2_, 5 mM EGTA, 50 mM imidazole, 0.2 mM GTP, and 1 mM β-mercaptoethanol; wash buffer 1:400 mM Pipes pH 6.8, 5 mM MgCl_2_, 5 mM EGTA, 0.2 mM GTP, 5 mM ATP, and 1 mM β-mercaptoethanol; wash buffer 2:400 mM Pipes pH 6.8, 5 mM MgCl_2_, 5 mM EGTA, 0.1% Tween-20, 10% glycerol, and 1 mM β-mercaptoethanol). Protein was eluted in 10 fractions of 0.5 mL using the elution buffer (400 mM Pipes pH 6.8, 5 mM MgCl_2_, 5 mM EGTA, 500 mM imidazole, 0.2 mM GTP, and 1 mM β-mercaptoethanol). The 10 fractions of protein were collected and pulled together to undergo buffer exchange in NMG buffer (10 mM sodium phosphate, pH 7.0, 0.5 mM MgCl_2_, and 0.1 mM GTP). The buffer exchange was performed in a 20 mL Amikon Ultra-4, 50K cut-off concentrator. Finally, the protein obtained was frozen in the presence of 0.25 mM trehalose in liquid nitrogen and stored at −80 °C.

### Crystallization and Crystal Structure Determination

For the T_2_R-TTL complex, tubulin (8 mg/mL), TTL (17 mg/mL), and RB3 (26 mg/mL) were mixed and concentrated (Amicon MWCO 10) at 4 °C to a final complex concentration of 20 mg/mL. The concentrated mixture was supplemented with 10 mM DTT, 0.1 mM GDP, and 1 mM AMPCPP before setting up crystallization experiments. Initial crystallization conditions were determined from previous structures^26^ using the sitting-drop vapor diffusion technique with a reservoir volume of 200 μL, a drop volume of 1 μL of complex, and 1 μL of reservoir solution at 20 °C. Crystal-producing conditions were further optimized using the hanging drop vapor diffusion method with a reservoir volume of 500 μL, a drop volume of 1 μL of complex, and 1 μL of reservoir solution. Native T_2_R-TTL complex was crystallized in 0.1 M Mes/0.1 M imidazole pH 6.5, 0.03 M CaCl_2_/0.03 M MgCl_2_, 5 mM l-tyrosine, 8.8% glycerol, and 5.5% PEG4000. Plates were kept at 20 °C and crystals appeared within the next 24 h. Suitable crystals were short-soaked (10–30 min) in reservoir solution containing 2 mM of PM534. Prior to flash-cooling in liquid nitrogen, crystals were cryo-protected using 10% PEG4000 and increasing glycerol concentrations (16 and 20%). X-ray diffraction data were collected on beamline BL13-XALOC (ALBA, Cerdanyola del Vallès, Spain). Diffraction intensities were indexed and integrated using XDS,^57^ and scaled using AIMLESS.^58^ Molecular replacement was performed with PHASER^59^ using the previously determined structure (PDB 4o2b) as a search model. Structures were completed with cycles of manual building in COOT^60^ and refinement in PHENIX.^61^ Data collection and refinement statistics are summarized in Table 1. Molecular graphics and analyses were performed with PyMol (The PyMol Molecular Graphics System, Version 2.3.2, Schrödinger, LLC) and COOT, respectively.

### Biochemistry

Tubulin polymerization assays were performed in 3.4 M glycerol, 10 mM phosphate, 1 mM EGTA, 6 mM MgCl_2_, 1 mM GTP pH 6.7 buffer at an 18 or 25 μM concentration of tubulin, as described.^62^

The binding constant of PM534 for tubulin was determined by competition with a well-characterized colchicine binding site ligand (R-PT), as described.^63^ The fluorescence emission of a mixed sample of 0.2 μM R-PT and 0.2 μM tubulin was evaluated in the presence of increasing concentrations of the studied ligand (0, 0.05, 0.2, 0.5, 2, 5, 10, 30, 50, and 70 μM). Samples were incubated for 30 min at 25 °C before the fluorescence emission intensity at 456 nm (excitation of 374 nm) was measured. The binding constant was obtained using Equigra V5.0.^64^

The dissociation constant of PM534 from the colchicine site in tubulin was measured in a photon-counting instrument Fluorolog 3-221 (Jobin Yvon-Spex, Longiumeau, France) with excitation and emission wavelengths of 365 and 430 nm, respectively. Upon adding 60 μM colchicine to a solution containing 2.5 μM tubulin in 10 mM Sodium Phosphate and 0.1 mM GTP, pH 7.0, preincubated for 30 min with 3 μM PM534, the dissociation of PM534 was assessed by the change in fluorescence intensity of the solution due to the binding of colchicine to the sites left empty by PM534. Adequate controls were performed to check the faster binding of colchicine compared with the dissociation of PM534.

### Cell Biology

Human NSCLC carcinoma-derived cells A549 and NCI-H460, human cervical carcinoma HeLa and HeLaβIII transfected cells,^42^ as well as human ovarian carcinoma A2780 and its P-gp overexpressing counterpart A2780AD^43^ cell lines were cultured at 37 °C in DMEM supplemented with 10% fetal calf serum, 2 mM l-glutamine, 1 mM pyruvate, 40 μg/mL gentamycin, 100 IU/ml penicillin and 100 μg/mL streptomycin in a 5% CO_2_ air atmosphere. Human lung adenocarcinoma NCI-H23 cell line was cultured in RPMI, while human lung anaplastic carcinoma Calu-6 cell line was cultured in MEME 10% fetal calf serum, both with the same supplements and at the same temperature and CO_2_ conditions as above. Antiproliferative assays were performed as described.^65^ Briefly, the MTT viability assay was used for the determination of the antiproliferative effect of PM534. Drugs were incubated for 48 h prior to the reaction. IC_50_ values were obtained by fitting the experimental data to a four-parameter logistic curve using SigmaPlot 14.5 software package (Systat Software, Inc., San Jose, CA, USA). For GI_50_ determination, cells were seeded in 96-well plates and cultured 24 h in drug-free medium prior to a 72 h treatment with the appropriate amounts of each compound (PM534, colchicine, or vinblastine). Dose–response curves and GI_50_s were calculated with GraphPad Prism v9.0 software.

Indirect immunofluorescence images were obtained using A549 cells plated at a density of 50,000 cells/ml onto 18 mm round coverslips, cultured overnight, and treated with increasing amounts of PM534, colchicine, podophyllotoxin, or the drug vehicle (DMSO) for 24 h. DMSO was always less than 0.5%. Cells were permeabilized using PEG and Triton X-100 and fixed with 3.7% formaldehyde, as previously described.^66^ Cells were incubated with a DM1A mouse monoclonal antibody (Sigma-Aldrich) reacting with α-tubulin. After that, samples were washed and incubated with AF488 goat antimouse polyclonal antibody (ThermoFisher), and 3 μM DAPI (Merk) was added for staining the DNA. Images were taken using a Leica DM 6000 B epifluorescence microscope employing a 100X objective with a N.A. of 1.46. Images of α-tubulin and DNA were usually taken at slightly different but close z planes in order to improve the clarity of the focus for each individual image. Images were recorded using a Leica DFC360 FX CCD camera.

### In Mice Xenograph Model

H460 cell line consists of a large human cell lung carcinoma originally obtained from the American Collection of Cell Cultures (ATCC HTB-177TM). Cells were maintained in vitro at 37 °C with 5% CO_2_ in DMEM. Culture cells were passaged every 3 to 5 days upon reaching confluence. Female athymic nu/nu mice between 4 and 6 weeks of age were purchased from Envigo (Barcelona, Spain). Animals were housed in individually ventilated cages (Sealsafe Plus, Techniplast S.P.A.), on a 12 h light–dark cycle at 21–23 °C and 40–60% humidity. Mice were allowed free access to an irradiated standard rodent diet (Tecklad 2914C) and sterilized water. Animals were acclimated for 5 days prior to being individually tattoo-identified and subcutaneously implanted with 5 × 10^6^ H460 cells suspended in 0.05 mL of 50% Matrigel (Corning Incorporated Life Sciences) and 50% Dulbecco′s Modified Eagle’s Medium (Sigma-Aldrich, Co) consisting solution without serum or antibiotics.

When tumors reached ca. 200 mm^3^, tumor-bearing animals were randomly allocated into the experimental groups (*N* = 6/group): PM534 was administered at 2.5, 1.7, 1.1, or 0.75 mg/kg and placebo. All treatments were intravenously administered once a week for two consecutive weeks (days 0 and 7). Tumor measurements were determined by using digital calipers (Fowler Sylvac, S235PAT). The formula to estimate tumor volume (mm^3^) from 2-dimensional tumor measurements was: tumor volume = (*a* × *b*^2^)/2, where, *a*: length (longest diameter) and *b*: width (shortest diameter) in mm of a tumor. Tumor volume and animal’s body weight were measured 2–3 times per week starting from the first day of treatment (day 0). Treatment tolerability was assessed by monitoring body weight evolution, clinical signs of systemic toxicity, as well as evidence of local damage at the injection site. Treatments which produced >20% lethality and/or 20% net body weight loss were considered toxic. Animals were euthanized when their tumors reached ca. 2000 mm^3^ and/or severe necrosis was observed.

NCI-H460 tumor xenografts were removed from animals treated with placebo or with PM534 (at the highest dose, 2.5 mg/kg) 24 h postadministration and then dissected free, formalin-fixed, paraffin-embedded for Hoechst 33258 staining. Apoptotic induction was determined by the number of nuclear apoptotic figures Hoechst 33258 stained in the tumor slices. Briefly, Triton X-100 (0.5%) permeabilized sections were stained with Hoechst 33258 (Sigma-Aldrich) diluted 1:5000 in PBS, for 1 h, rinsed with water, mounted, and analyzed under a fluorescence microscope (λ_ex_ = 334 nm/λ_em_ = 465 nm). The number of apoptotic cell bodies was quantified by recording 5 high-power fields (magnification 40×), and samples were evaluated using Cell Olympus software (v3.3, Olympus, Japan) by a blinded independent pathologist. Animal protocols were reviewed and approved according to the regional Institutional Animal Care and Use Committees (IACUC).

Design, randomization, and monitoring (including body weight and tumor measurements) were performed using NewLab Oncology Software (version 2.5, France). Tumor volume data from groups were compared using a two-tailed Mann–Whitney U test. The data was presented as medians. Survival statistical differences between groups were assessed by applying Kaplan–Meier curves with the log rank test. Statistical analysis and graphs were performed using GraphPad Prism (GraphPad Software Inc., San Diego, USA).

## Supporting Information Available

TOF-HRMS, chromatographic analysis, and NMR analysis and cross eye stereoscopic figure of PM534 in its tubulin binding site (PDF)
SMILES data (CSV)

## Accession Codes

The PDB code for the crystallographic structure of the T2RTTL-PM534 complex is 7ZYW.

## Abbreviations

CBD: colchicine binding domain
MDA: microtubule-destabilizing agent
MSA: microtubule-stabilizing agent
MTA: microtubule-targeting agent
MTC: 2-methoxy-5-(2,3,4-trimethoxyphenyl)-2,4,6-cycloheptatrien-1-one
NSCLC: non-small cell lung cancer
P-gp: P-glycoprotein
R-PT: (R)-(+)-ethyl 5-amino 2-methyl-1,2-dihydro-3-phenylpyrido[3,4-*b*]pyrazin-7-yl carbamate.
