# Supplementary material for: PM534, an Optimized Target-Protein Interaction Strategy through the Colchicine Site of Tubulin
Source: J Med Chem. 2024 Jan 31;67(4):2619–30. doi: 10.1021/acs.jmedchem.3c01775 (PMC10895673; doi:10.1021/acs.jmedchem.3c01775)

SUPPORTING INFORMATION

PM534, an optimized target protein interaction strategy through the colchicine site of tubulin

Daniel Lucena-Agell[1], María José Guillén[2], Ruth Matesanz[1], Beatriz Álvarez-Bernad[1], Rafael Hortigüela[1], Pablo Avilés[2], Marta Martínez-Díez[2], Gema Santamaría-Núñez[2], Julia Contreras[3], Iván Plaza-Menacho[3], Juan F. Giménez-Abián[1], María A. Oliva[1], Carmen Cuevas[2*] and J. Fernando Díaz[1*]

[1]Centro de Investigaciones Biológicas Margarita Salas, Consejo Superior de Investigaciones Científicas, Ramiro de Maeztu 9, 28040 Madrid, Spain.

[2]PharmaMar S.A., Avda de los Reyes 1, 28770 Colmenar Viejo, Madrid, Spain.

[3]Centro Nacional de Investigaciones Oncológicas (CNIO), Melchor Fernández Almagro 3, 28029 Madrid, Spain.

*Corresponding Authors.

Corresponding authors e-mail address: ccuevas@pharmamar.com ; fer@cib.csic.es

Contents of SI

-Purity analysis of PM534

-Cross eye stereoscopic figure of PM534 in its tubulin binding site.

Purity Analysis of PM534

1.-TOF-HRMS

A sample of **PM534** active substance was analyzed by Time-Of-Flight-High Resolution Mass Spectrometry (TOF-HRMS). The results of TOF-HRMS in **Supplementary Table 1** are consistent with the expected molecular weight and elemental composition for **PM534**.

Supplementary Table 1: TOF-HRMS of **PM534**

**Molecular Formula** (protonated)	**Assignment**	**Theoretical Value**	**Experimental Value**
$C_{20}H_{28}N_3O_5S$	$[M+H]^+$	422.1744	422.1774

2.-Chromatographic analysis.

PM534 content as well as the impurities in the active substance are determined by a validated selective and sensitive linear gradient HPLC method with DAD/UV detection.

2.1.- Chromatographic Conditions

The critical method parameters presented in **Supplementary Table 2** may vary slightly (based on scientific grounds) provided that the system suitability requirements are met and comparable separation of all relevant compounds is demonstrated.

Supplementary Table 2: HPLC chromatographic conditions (assay and impurities).

Parameter	Description
**Column**	Column: UltraCore Super C18, 150 mm x 4.6 mm, 2.5 µm<br>Pre-Column Filter: UHPLC
**Oven Temperature**	+60 ºC
**Auto Sampler Temperature**	+20 ºC
**Detector**	DAD: λ = 280 nm (BW: 8 nm), λ_{Ref} = 380 nm (BW: 40 nm)<br>UV: λ = 280 nm
**Detection Time**	60 minutes
**Mobile Phase**	**A:** Water<br>**B:** Acetonitrile
**Elution**	Linear Gradient Program: (see below)
**Flow Rate**	1.0 mL/min
**Injection Volume**	20 µL
**Injector Needle Wash**	Acetonitrile
**Run Time**	70 minutes
**PM534 Retention Time**	Approximately 32 minutes
**Sample Preparation**	Standard and sample solutions contain 0.5 mg/mL of PM534

Elution – Linear Gradient Program:

Time (min)	**Mobile Phase A (%)**	**Mobile Phase B (%)**
0	85	15
10	70	30
40	60	40
50	0	100
60	0	100
61	85	15
70	85	15

Full scale and enhanced representative chromatograms of **PM534** and impurities obtained under these conditions are provided in **Supplementary Figure 1** and **Supplementary Figure 2**, respectively.

Supplementary Figure 1: Full scale representative chromatogram of **PM534** and impurities.

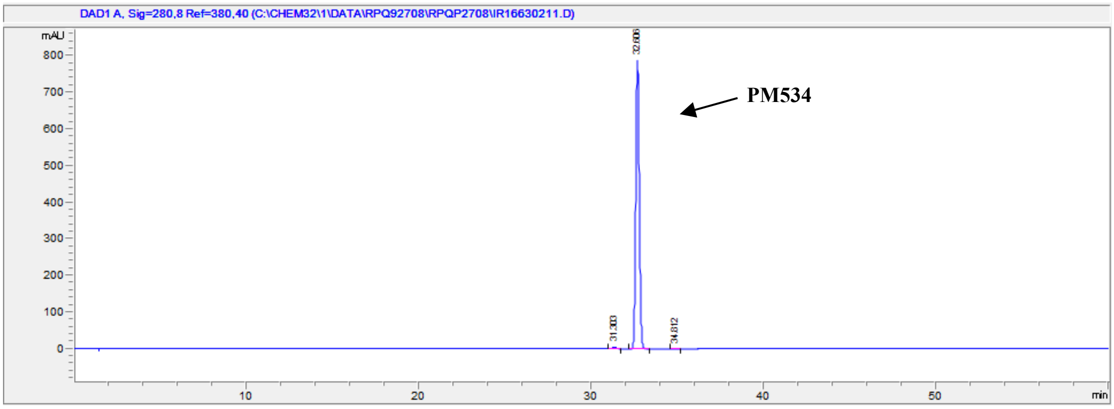

Supplementary Figure 2: Enhanced representative chromatogram of **PM534** and impurities.

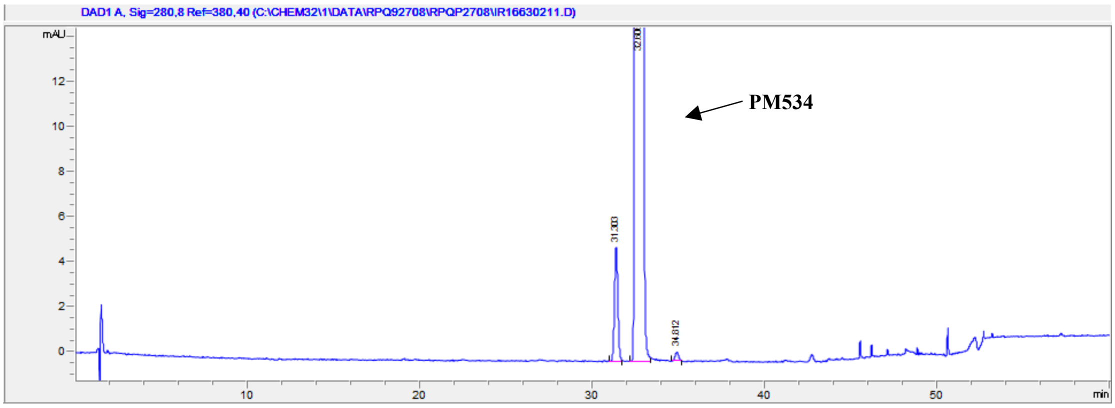

3.- NMR analysis.

The proton nuclear magnetic resonance (^{1}H-NMR) and carbon nuclear magnetic resonance (^{13}C-NMR) experiments were performed using an appropriate amount of **PM534** active substance in CD_3OH (Sigma-Aldrich) solution.

The 500 MHz ^{1}H-NMR and 125 MHz ^{13}C-NMR spectra are shown in **Supplementary Figures 3 and 4**. respectively.

Full assignment of proton and carbon chemical shifts are listed in **Supplementary table 3**.

Supplementary Table 3: NMR data of PM534 in CD_3OH (500 MHz for ^1H and 125 MHz for ^{13}C).

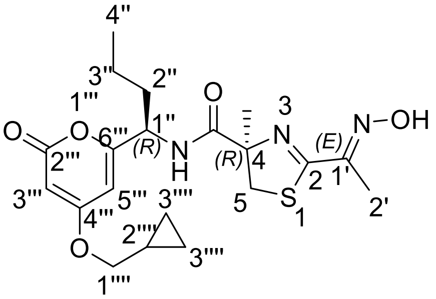

Position/Structure	PM534	
	**¹H-NMR** (ppm)	**¹³C-NMR** (ppm)
	**δ** [a], **m** [b], ***J*** (Hz) [c]	**δ** [a]
2	-	170.1
4	-	85.5
C^4-Me	1.52, s	24.8
5	3.55, d (11.6)<br>3.17, d (11.5)	40.4
C=O	-	176.4
1’	-	152.8
2’	2.18, s	10.9
1’’	4.78, ddd (8.9, 8.9, 5.7)	52.1
2’’	1.88, m<br>1.81, m	35.1
3’’	1.47, m<br>1.39, m	20.1
4’’	0.98, t (7.4)	13.7
2’’’	-	166.7
3’’’	5.48, s	89.1
4’’’	-	172.5
5’’’	6.05, d (2.6)	100.9
6’’’	-	165.1
1’’’’	3.88, d (10.8)<br>3.84, d (10.8)	75.2
2’’’’	1.23, m	10.2
3’’’’	0.63, m, 2H<br>0.35, q (5.0), 2H	3.5
NH	7.87, d (8.6)	-

δ [a]: chemicals shifts are reported in ppm using residual methanol (3.34 for 1H and 49.9 for 13C) as internal reference.

m [b]: multiplicity.

J [c]: coupling constants are reported in Hz.

s = singlet; d = doublet; t = triplet; q = quartet; m = multiplet.

Supplementary Figure 3: ^{1}H-NMR of **PM534** in CD_3OH (500 MHz).

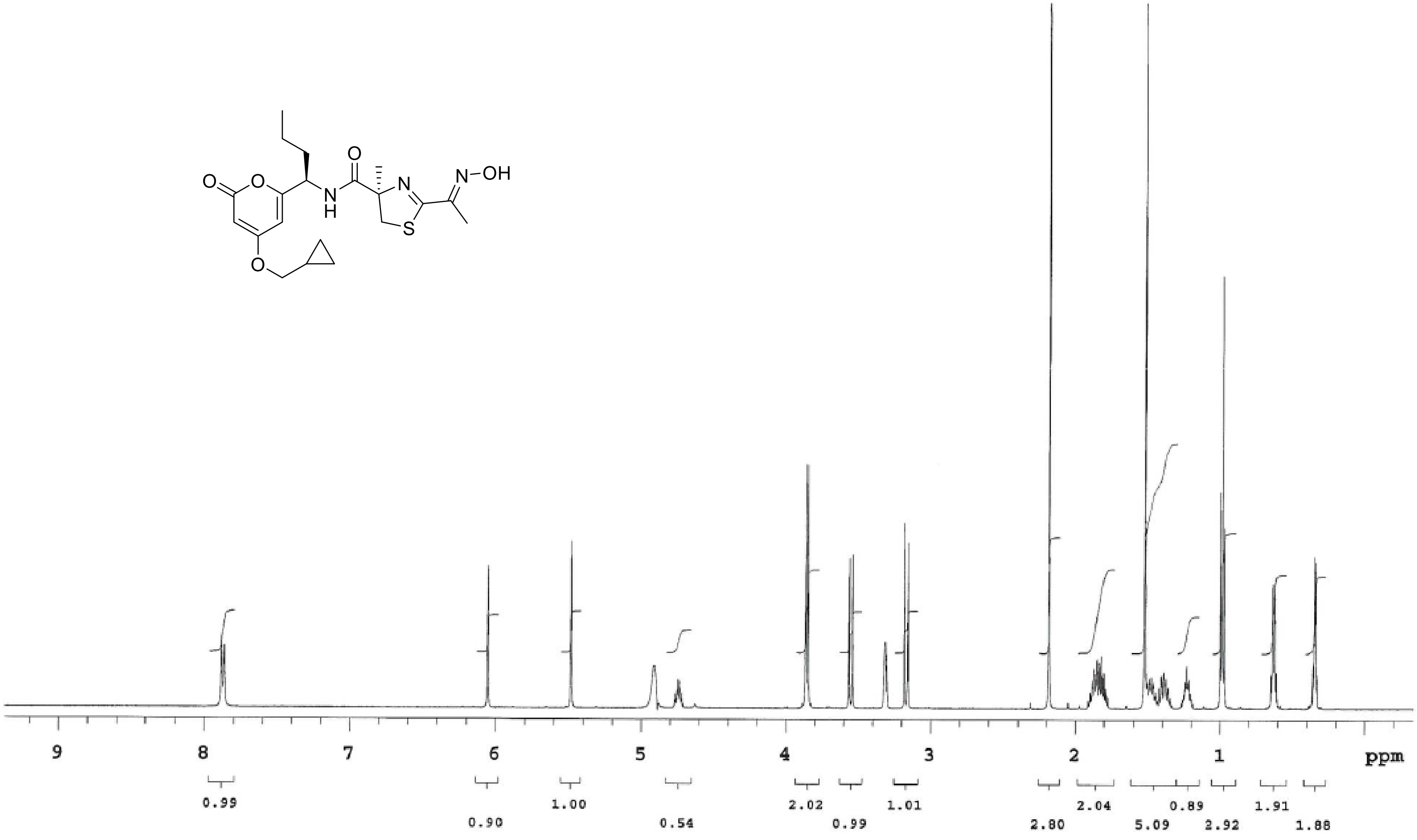

Supplementary Figure 4: ^{13}C-NMR of **PM534** in CD_3OH (125 MHz).

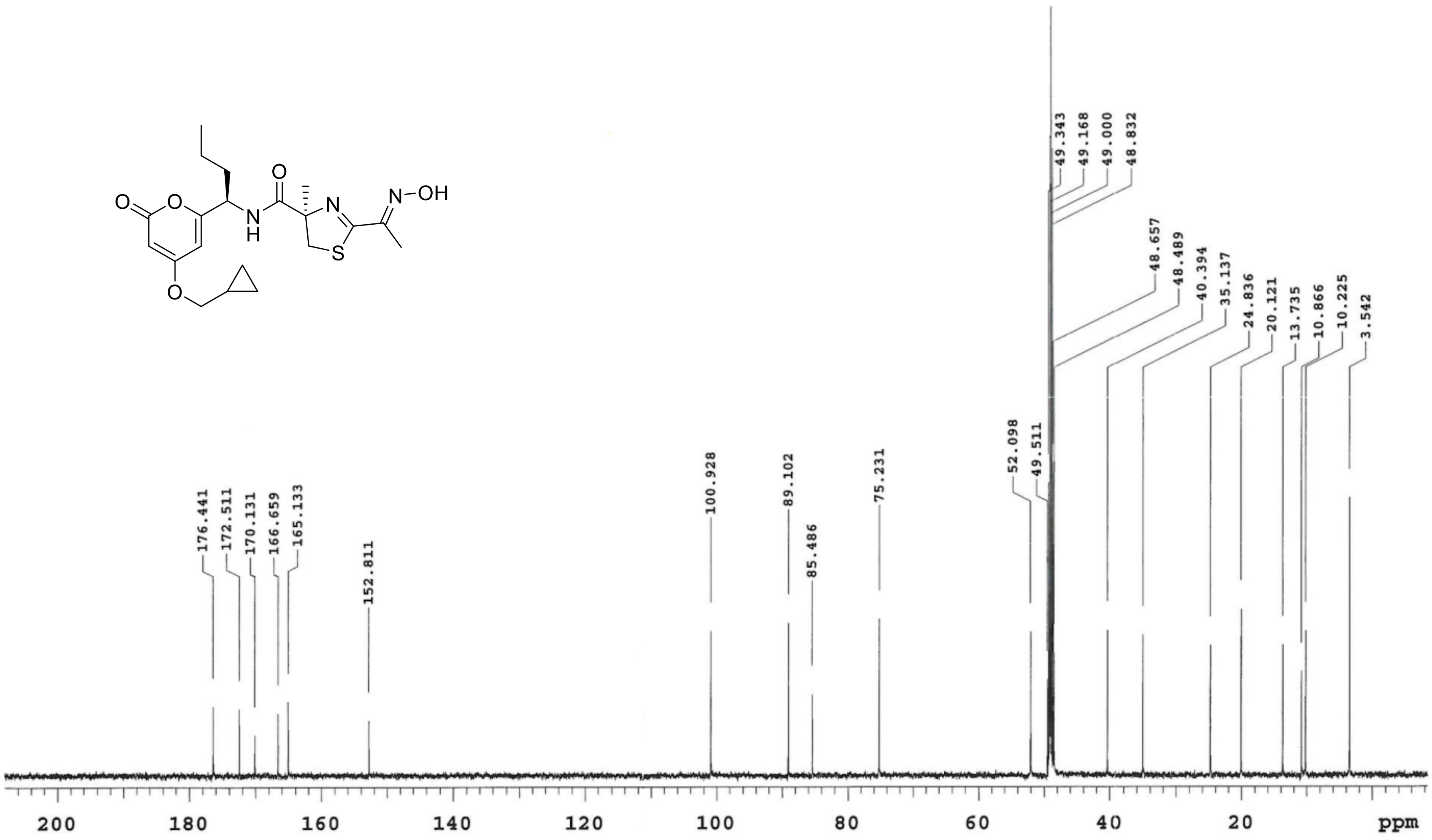

Supplementary Figure 5.- Cross eyed stereo zoom into the colchicine domain in β-tubulin (ribbon representation chain B in white) PM534 (sticks salmon).

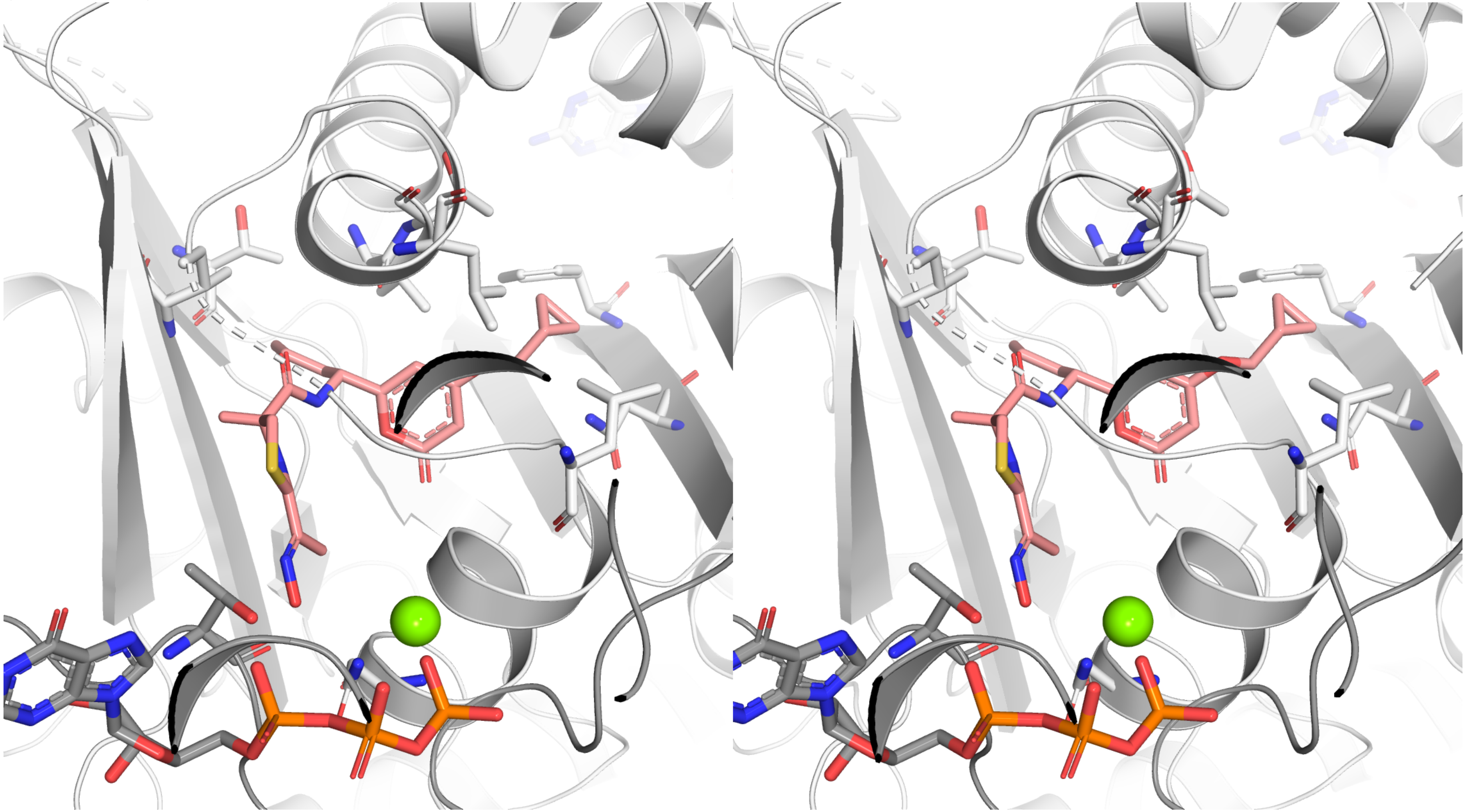

Supplement: Supplementary file 1 — jm3c01775_si_001.pdf [file jm3c01775_si_001.pdf]
